# Time of injury affects urinary biomarker predictive values for acute kidney injury in critically ill, non-septic patients

**DOI:** 10.1186/1471-2369-14-273

**Published:** 2013-12-09

**Authors:** Hilde RH de Geus, Gijs Fortrie, Michiel GH Betjes, Ron HN van Schaik, AB Johan Groeneveld

**Affiliations:** 1 Department of Intensive Care, Erasmus University Medical Center, Rotterdam, the Netherlands; 2 Department of Nephrology, Erasmus University Medical Center, Rotterdam, the Netherlands; 3 Department of Clinical Chemistry, Erasmus University Medical Center, Rotterdam, the Netherlands; 4 Department of Intensive Care Medicine, Erasmus University Medical Center, H619 PO box 2040, Rotterdam, CA 3000, The Netherlands

**Keywords:** Urinary biomarkers, AKI, NGAL, KIM-1, Pi-GST and alpha GST

## Abstract

**Background:**

The predictive value of acute kidney injury (AKI) urinary biomarkers may depend on the time interval following tubular injury, thereby explaining in part the heterogeneous performance of these markers that has been reported in the literature. We studied the influence of timing on the predictive values of tubular proteins, measured before the rise of serum creatinine (SCr) in critically ill, non-septic patients.

**Methods:**

Seven hundred adult critically ill patients were prospectively included for urine measurements at four time-points prior to the rise in serum creatinine (T = 0, -16, -20 and -24 h). Patients with sepsis and or AKI at ICU entry were excluded. The urinary excretion of the proteins, neutrophil gelatinase-associated lipocalin (NGAL) and kidney injury molecule-1 (KIM-1), which are up-regulated in the distal and proximal tubules, respectively, were measured as well as the constitutive cytoplasmatic enzymes, π- and α-glutathione-S-transferase (GST), which are released by the distal and proximal tubules, respectively.

**Results:**

Five hundred and forty-three subjects were eligible for further analyses; however, 49 developed AKI in the first 48 h. Both NGAL (P = 0.001 at T = -24 vs. non-AKI patients) and KIM-1 (P < 0.0001 at T = 0 vs. non-AKI patients) concentrations gradually increased until AKI diagnosis, whereas π- and α-GST peaked at T = -24 before AKI (P = 0.006 and P = 0.002, respectively vs. non-AKI patients) and showed a rapid decline afterwards. The predictive values at T = -24 prior to AKI were modest for π- and α-GST, whereas NGAL sufficiently predicted AKI at T = -24 and its predictive power improved as the time interval to AKI presentation decreased (area under the receiver operating characteristic curve; AUC = 0.79, P < 0.0001). KIM-1 was a good discriminator at T = 0 only (AUC = 0.73, P < 0.0001).

**Conclusions:**

NGAL, KIM-1, pi- and alpha-GST displayed unique and mutually incomparable time dependent characteristics during the development of non-sepsis related AKI. Therefore, the time-relationship between the biomarker measurements and the injurious event influences the individual test results.

## Background

There is an on-going search for biomarkers for AKI prediction. These biomarkers, may help, in the future, to guide preventive and therapeutic measures to benefit patients [1-13]. The AKI-induced up regulation of low molecular weight proteins, such as neutrophil gelatinase-associated lipocalin (NGAL) and kidney injury molecule-1 (KIM-1), and their subsequent release and excretion into the urine have been studied in AKI patients and patients who are at risk for the condition [6-9,11,13-18]. Currently, NGAL, presumably from distal tubular origins at least in experimental AKI [19], is the most frequently described human AKI biomarker, although it is not perfect, and NGAL is considered as a reference standard [3,5,6,8-13,20,21]. Nevertheless, the literature possesses a marked heterogeneity in its reported AKI predictive power. The clinical value of KIM-1, a predominantly ischaemic proximal tubular injury marker [1,4,5], remains uncertain, with reports suggesting superiority [1,4] or inferiority [3,5,13] compared with other markers. The constitutive cytoplasmatic enzymes, π- and α-glutathione-S-transferase (GST), are detectable in the urine when the cell wall integrity of the distal and the proximal tubules are damaged, respectively [22]. However, the few clinical studies regarding the AKI predictive value of these enzymatic markers are conflicting [2,3,9,23] and the only comparison evaluating urinary NGAL was limited to a post cardiac surgery study [9]. Additionally, the available literature reports heterogeneous predictive performances for AKI biomarkers in different patient populations and conditions, such as adult vs. pediatric patients, sepsis vs. non-sepsis states, surgical vs. non-surgical conditions, developing vs. established AKI and fixed vs. non-fixed intervals between injury and sampling.

In this study we aimed to evaluate the predictive performance variation of urinary AKI biomarkers that precede the rise in serum creatinine (SCr). Additionally, we sought to study their individual kinetics as a function of time in non-septic patients, because predictive values and optimal cut-off levels for AKI markers may differ between septic and non-septic AKI [24]. In contrast to our previous study, which included AKI at ICU entry [11], the primary endpoint for the current study was AKI development within 48 h following ICU admission. Indeed, AKI prediction is more useful than confirming established AKI; however, many previous studies grouped together developing and established AKI, thereby potentially leading to predictive value overestimation [1-6,8-10,12,13,21].

## Methods

### Setting

This was a prospective single centre cohort study in a 30-bed closed format university hospital intensive care unit (ICU) in which general surgical, trauma, medical, neurological and neurosurgical, but not cardiac surgery, patients were treated. All consecutively admitted adult critically ill patients, between September 1, 2007, and April 1, 2008, were considered eligible. Exclusion criteria included the following: patients under 18 years of age, readmissions during the inclusion period, refusal of informed consent, a history of nephrectomy, documented chronic kidney disease (CKD) (>stage 3) or kidney transplantation and a sepsis diagnosis at the time of ICU entry. Sepsis and CKD were applied as exclusion criteria to avoid their confounding roles in biomarker expression. The study was approved by the Erasmus MC University Medical Centre Institutional review board (Rotterdam, the Netherlands). Deferred patient consent was used in combination with written informed consent that was obtained from the participants or their health care proxy within 48 h following ICU admission. In the consent refusal cases (n = 6, 0.9%), the collected urine specimens were appropriately destroyed. This study was a sub-study of a previously reported study [11].

### Protocol, sample collection and processing

Demographic data were recorded, including the severity of illness scores, several renal and outcome parameters (such as the hospital discharge serum creatinine levels), the duration of ICU stay and the 28-day and in-hospital mortality rates. Serum creatinine values were available at the time of admission and at 6:00 am daily thereafter, until 72 h after entry. AKI diagnoses were approximated and calculated during admission and as close as possible 24 and 48 hours after admission. The serum creatinine levels were measured in the hospital’s clinical chemical laboratory with a Roche enzymatic kit, (which provided similar results to a well-regarded reference method) based on isotope dilution mass spectrometry. Urinary output and fluid balances were also recorded. At ICU admission (T = 0), and at T = 4, 8, 24 hrs thereafter, urine samples were collected using a urine catheter. The samples were processed in the hospital’s laboratory and the supernatants were stored at -80°C. NGAL (Triage® immunoassay, Biosite Inc. Alere, San Diego, CA, USA), KIM-1, π- GST, and α-GST (Argutus Medical, Dublin, Ireland) concentrations were measured using research-based immunoassays. The detection limits for the urine NGAL assays were 2.6 - 4100 ng/ml. The assays’ coefficient of variation was 13.9%. The π- GST, α-GST and KIM-1 assay detection limits were 3.12-100 ng/ml, 6.25-200 ng/ml and 0–10 ng/ml, respectively. These assays average coefficient of variation in this study were 4%, 3% and 1%, respectively.

### Definitions

The baseline serum creatinine levels were defined as the steady state levels four weeks to six months prior to ICU admission. If these values were not available, the admission value was applied as the baseline. The sepsis criteria were a clinically suspected or confirmed infection, a temperature above 38.5°C or below 36.0°C, tachycardia (>90 beats/min) and tachypnea (>20/min) or necessity for mechanical ventilation, and leukocytosis >12 ×10^9^/L or >10% bands, or leukopenia <4 ×10^9^/L. AKI was defined using the acute kidney injury network (AKIN) classification for serum creatinine changes relative to a steady state baseline value (AKIN 0 = no-AKI, AKIN 1 = serum creatinine increase >50% or an absolute serum creatinine rise of 0.3 mg/dL (=26.5 μmol/L) compared to baseline, AKIN 2 = serum creatinine increase >100% and AKIN 3 = serum creatinine increase >200%) without using the urine output (UP) criteria. To plot the biomarker expression levels that preceded AKI, the time-points following ICU admission were recoded as the time-points preceding AKI. AKI occurred either at T = 24 or T = 48 following ICU admission. The initial AKI time-point was recoded as T = 0 and the available measurements preceding this time point were recoded relative to T = 0 (including T = -48, T = -44, T = -40, T = -24, T = -20 and T = -16 hrs). Fifty-six patients had more than one ICU admission; therefore, only the data from their first admission were used.

### Statistical analysis

Patients were grouped according to whether they lacked AKI or had a developing AKI within the first 48 h of admission. Most continuous data were distributed non-normally (Kolmogorov-Smirnov test P < 0.05). We compared developing AKI patients and non-AKI patients using univariate analyses for continuous variables (Mann–Whitney U test) and categorical variables (using the χ^2^ or Fisher exact test). Two-tailed tests were used throughout. Receiver operating characteristics curve (ROC) analyses were used to assess the predictive value of biomarkers in developing AKI patients. The area under the curve (AUC), with 95% confidence intervals [95% CI], was calculated and compared. Statistical analyses were performed with the SPSS statistical software package, version 16.0 (SPSS, Chicago, IL, USA) for windows as well as MedCalc for Windows, version 9.5.0.0 (MedCalc Software, Mariakerke, Belgium). The data were reported as numbers (percentages) or as medians (with interquartile ranges), where appropriate. Means and standard errors of the mean (SEM), however, were used in the time course graphs for the sake of clarity. A P ≤ 0.05 was considered statistically significant, and exact values are presented throughout.

## Results

### Patient characteristics

**Figure 1:**
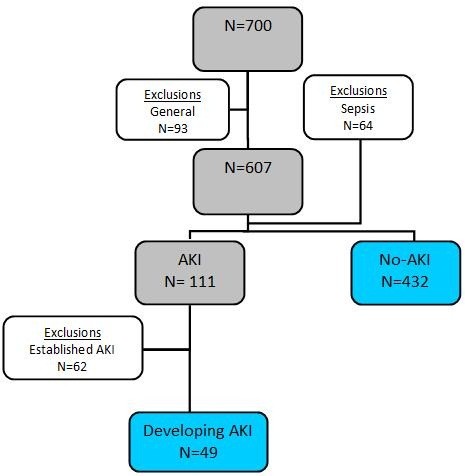
**Study cohort flow chart. ***N:* number; *AKI:* acute kidney injury; *established AKI:* AKI at the time of ICU admission and *developing AKI:* AKI developing at or 24 hours following admission.

Seven hundred consecutive ICU admissions were included in the study. Six patients refused consent (0.9%), 6 patients had previously undergone a nephrectomy (0.9%), 56 admissions were counted as readmissions during the study period (8%) and 25 patients had chronic kidney disease (CKD) stage 3 or a kidney transplant (4%). Of the remaining 607 patients, 64 subjects were diagnosed with sepsis; therefore these patients were also excluded, leaving 543 cases for the final analysis. Out of the 111 patients with AKI within the first 48 hours of admission, 62 of these patients (56%) had already met the AKI criteria at the time of ICU entry, leaving 49 developing AKI subjects (Figure 1). The admission diagnoses were subdivided into non-cardiac postoperative (N = 185), respiratory insufficiency (N = 110), subarachnoid or intracerebral bleeding (N = 99), multi-trauma (N = 42), isolated neurotrauma (N = 29), liver transplantation (N = 28), cardio pulmonary resuscitation (N = 26), haemorrhagic shock (N = 20), multi organ failure (N = 2) and lung transplantation cases (N = 1) with one missing diagnosis.

The developing AKI patients were older, more severely ill and more often male (Table 1). Furthermore, the developing AKI patients had higher pre-admission baseline serum creatinine levels and a higher cumulative fluid balance within the first 24 h of ICU admission. At hospital discharge, SCr values were higher in patients who had an AKI episode compared with the non-AKI patients. Additionally, the 28-day and hospital mortality rates were higher as well in the AKI patients.

**Table 1:** Patient characteristics

	**Non-AKI**	**Developing AKI**	**P**
	**(N = 432)**	**(N = 49)**	
Age, years	57(25)	61(25)	0.04
Gender male, n (%)	243(56)	38(77)	0.004
BMI (kg/m^2^)	24.6(4.8)	25.1(4.4)	0.67
APACHE II	16(9)	23(11)	<0.001
SOFA	4(4)	9(6)	<0.001
Admission diagnosis, n (%)			
Medical	100(23)	18(36)	0.11
Surgical	215(49)	20(40)	
Neurological	117(27)	11(22)	
*Renal characteristics*			
Baseline SCr (mg/dl)	0.74(0.3)	0.85(0.3)	0.002
UP (ml/kg/h)	1.1(0.8)	0.9(0.9)	0.32
FB (l)	1.9(3.0)	4.3(4.4)	<0.001
AKIN-stages, n (%)			
AKIN-1		34	
AKIN-2		11	
AKIN-3		4	
Patients with CVVH, n (%)		3(6)	
*Outcome*			
SCr at hospital discharge (mg/dl)	0.68(0.2)	0.77(0.6)	0.002
ICU days	3(5)	7(11)	<0.001
28-day mortality (%)	53(12)	15(30)	0.002
Hospital mortality (%)	59(13)	17(34)	0.001

*Abbreviations*: AKI: acute kidney injury; BMI: body mass index: APACHE II: Acute physiology and chronic health evaluation score; SOFA score: sequential organ failure assessment score; UP: urine production in 24 h after admission per kg ideal body weight; FB: fluid balance in 24 h; CVVH: continuous veno-venous haemofiltration; ICU: intensive care unit. Median (IQR) or number of patients (percentage) where appropriate.

### The biomarker patterns following ICU admission

**Figure 2:**
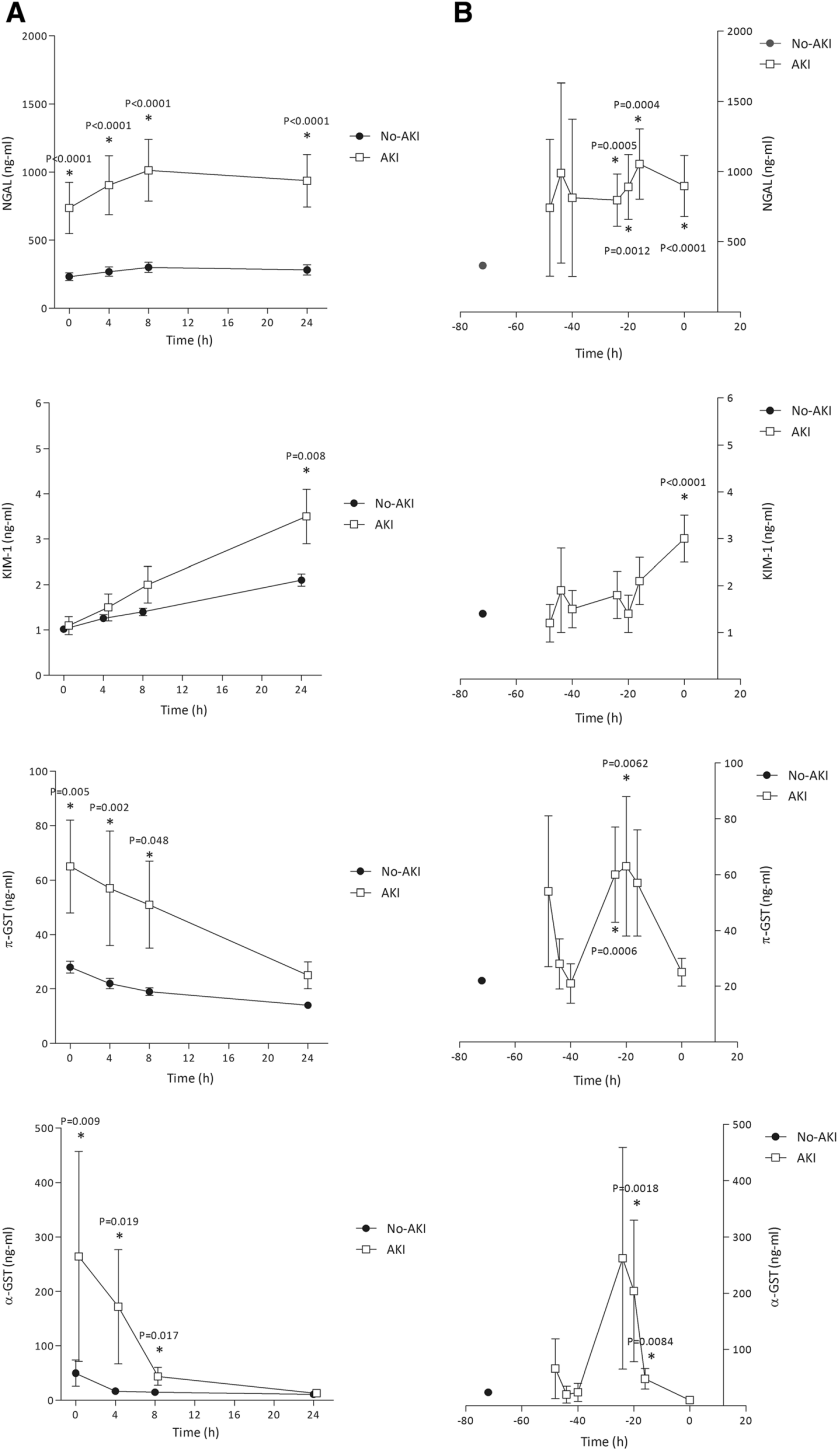
**Biomarker patterns after ICU admission (A) and preceding AKI (B).** Biomarker concentrations are expressed in ng/ml and data represent the mean (standard error of the mean; SEM). *NGAL*: Neutrophil gelatinase-associated lipocalin; *KIM-1*: Kidney injury molecule-1; *GST:* glutathione-S-transferase; *AKI*: acute kidney injury. Mean biomarker concentrations in AKI patients vs. non-AKI patients at each time-point were compared using the Mann–Whitney U test **(A)** and the mean biomarker concentrations in AKI patients were compared to the pooled mean value of all available non-AKI measurements using the Mann–Whitney U test **(B)**. Panel **A** represents the un-recoded data plotted against the time following ICU admission. Panel **B** represents the recoded data prior to the rise in SCr.

The biomarker levels following ICU admission for the developing AKI patients and those without AKI are shown in Figure 2A. This panel represents the not-recoded data. The upregulated NGAL and KIM-1 protein concentrations increased over the time following ICU admission, whereby NGAL increased right from the time of admission (P < 0.0001); the KIM-1 levels differentiated between the non-AKI and AKI at the T = 24 hour time point for the first time (P = 0.008). The KIM-1 concentrations in the non-AKI patients increased over the time following ICU admission. The constitutive enzyme concentrations, π- and α-GST, both decreased but remained higher up until 8 hours after admission in the AKI compared to the non-AKI patients (P ≤ 0.048 and P ≤ 0.017 respectively).

### The biomarker patterns preceding AKI

Figure 2B shows the pre-AKI biomarker patterns. All available non-AKI biomarker values were pooled to represent the non-AKI concentration in the graph represented at T = -72. The upregulated proteins KIM-1 and NGAL gradual increased in concentration prior to the SCr increase. KIM-1, however, was different in the AKI patients compared with the non-AKI patients right at the time of AKI presentation (T = 0, P < 0.0001). This contrasted with NGAL, which displayed a quicker response with different concentrations in the AKI compared with the non-AKI patients, starting at 24 hours prior to the AKI presentation time (P = 0.0005). The constitutive enzyme concentrations, π- and α-GST, peaked at 24 and 20 hours prior to the times SCr rose (T = 0), respectively, compared with the non-AKI patients (P = 0.006 and P = 0.0018). After a sudden peak, the biomarker concentrations declined quickly prior to AKI presentation times.

### AKI prediction

Table 2 shows the area under de curves (AUC’s) for the prediction of developing AKI for each individual biomarker at the different time points. NGAL displayed the most consistent predictive performance, starting 24 hours prior to AKI presentation (AUC = 0.66, P = 0.0005) and increased closer to the AKI endpoint (AUC = 0.79, P < 0.0001). In contrast, KIM-1 only “predicted” AKI at the same time when the rise in SCr levels occurred for the first time (AUC = 0.73 P < 0.0001). However, the π- and α-GST predictive power was modest (AUC = 0.65 for both) even at their peak concentrations (24 and 20 hours prior to AKI, respectively).

**Table 2:** ROC curves for developing AKI predictions vs. non-AKI patients

**Biomarker**	**Time**	**AUC (95% CI)**	**P**
NGAL	T = -24	0.66 (0.57-0.75)	0.0005
	T = -20	0.66 (0.57-0.75)	0.001
	T = -16	0.68 (0.57-0.78)	0.0004
	T = 0	0.79 (0.73-0.85)	<0.0001
KIM-1	T = 0	0.73 (0.64-0.83)	<0.0001
π-GST	T = -24	0.65 (0.56-0.75)	0.0006
	T = -20	0.64 (0.54-0.73)	0.006
α-GST	T = -20	0.65 (0.56-0.75)	0.002

*Abbreviations*: ROC: receiver operating characteristics curve; AKI: acute kidney injury; GST: glutathione-s- transferase, KIM-1: kidney injury molecule 1 and NGAL: neutrophil gelatinase associated lipocalin.

## Discussion

The present study shows that NGAL, KIM-1, pi- and alpha-GST show unique and mutually incomparable time dependent characteristics during the development of non-sepsis related AKI. The time-relationships between the biomarker measurements and the injurious renal hit therefore influenced the individual predictive test results. The constitutive enzymes displayed a narrow time window of expression, whereas NGAL outperformed KIM-1 in its early expression levels prior to an AKI diagnosis.

The NGAL predictive value for (non-septic) AKI in this study is in accordance with other work [3,5,6,8-13,20,21,25]. The expression pattern of NGAL prior to the rise in SCr is early and its predictive power also increases closer to the AKI presentation time. This latter observation suggests that the time-to-injury relationship is important and should be obtained for a correct interpretation of its AKI predictive value. KIM-1’s expression was less accurate and late (in relation to the time of SCr increase) compared with NGAL in the current study. This confirms the work by others who also evaluated adult critically ill patients [1,3,4,7,9]. Moreover, the predictive value of KIM-1, which only slowly increased in the time following renal injury, as our data suggest, is higher if the AKI has already developed, as in cardiac surgery, rather than if the AKI develops over the course of time, as in our study [1,4,5,9]. The rise in KIM-1 over time even when AKI (defined as a rise in SCr) does not develop can perhaps be explained by subclinical injury, because KIM-1 is a transmembrane glycoprotein exclusively present in the epithelial cells that survive after injury and facilitate necrotic cell debris phagocytosis [1,9]. This may look, at first sight, like a clinically irrelevant observation without direct implications. We believe, however, that this might imply the loss of renal reserve, which will most likely become relevant when the kidney suffers new injurious hits.

π- and α-GST were only modest AKI predictors in this population with slightly better or similar results compared with those reported in adult cardiac surgery patients (AUC = 0.54 [95% CI 0.42-0.66]) [9,26]. These data, however, were similar in another cohort of general critically ill patients [25]. In an older study, the markers were suggested to be superior to other enzymes AUC = 0.93 [0.74-0.99] and 0.89 [0.69-0.98] respectively [23]; however, these results were not reproduced in subsequent studies. Several other studies described their diagnostic performances in established AKI [2,26,27]. These results, however, are incomparable to the present data for these enzymatic markers, the sampling time in relation to the injurious hit seems to be especially critical for their ability to predict a rise in SCr at a later time point. This might make this category of biomarkers less well applicable in patients without a circumscribed time point of renal injury, such as is the case in general ICU patients. However, due to their sudden urine concentration changes, their applicability might be more appropriate in a setting of that monitors the renal toxic effects of drugs and contrast agents in the kidneys.

There are several limitations to the current results. Despite the large initial number of included patients, the developing AKI patient subset was relatively small, because 64% of the AKI patients had AKI at ICU entry and were thus excluded from the current analysis. The subset of patients with developing septic AKI was even smaller and did not allow for sufficient data analyses, although it would have been interesting to study the possible differences in biomarker expression between both septic related and non-septic related AKI. Despite the recognition that serum creatinine is a poor indicator of renal injury, (based on its varying tubular secretion levels among other reasons), it is still used in many studies [3,12,28]. Therefore, the usefulness of potentially more sensitive markers might be underestimated. We believe this phenomenon is reflected by our data, which indicate the presence of subclinical tubular injury in non-AKI patients (i.e., the increase in KIM-1 levels in non-AKI patients according to the AKIN classification). Urinary biomarkers can be used in non-anuric AKI only, therefore, narrowing their clinical applicability. Controversy exists on whether the correction for urinary creatinine concentrations is necessary for the interpretation of the results. We believe that normalisation to urine creatinine concentration poses a unique limitation because AKI patients are not in a steady state of creatinine turnover. Furthermore, several authors have shown that this effort does not contribute much to the final outcomes [1,2,5,6,9,13,23].

## Conclusions

Our current data suggest that the different biomarker expression pattrens, such as upregulated proteins and constitutive enzymes, and the time of sampling with respect to the actual time of cellular injury may partially explain the previously observed predictive value heterogeneity. These factors should be taken into account in future studies.

## Abbreviations

AKI: Acute kidney injury; SCr: Serum creatinine; NGAL: Neutrophil gelatinase associated lipocalin; KIM-1: Kidney injury molecule-1; GST: Glutathione-S-transferase; ICU: Intensive care unit; CKD: Chronic kidney disease; AKIN: Acute kidney injury network; ROC: Receiver operating characteristics curve; AUC: Area under the curve; CI: Confidence interval; SEM: Standard error of the mean.

## Competing interests

Hilde de Geus has received speaker fees from Alere. Alere and Argutus medical kindly provided the biomarker measurements. The other authors have nothing to declare.

## Authors’ contributions

HG conceived the study, participated in the design, created the database, performed statistical analyses and drafted the manuscript. GF assisted in additional data collection. MB participated in the study design and helped draft the manuscript. RS carried out sample processing and storage. JG participated in the study design, statistical analyses and the drafting of the manuscript. All authors read and approved the final manuscript.

## Pre-publication history

The pre-publication history for this paper can be accessed here:

http://www.biomedcentral.com/1471-2369/14/273/prepub
